# Traditional Chinese medicine on treating ureteral calculi: A systematic review and meta-analysis protocol

**DOI:** 10.1097/MD.0000000000017057

**Published:** 2019-09-13

**Authors:** Haisong Li, Sheng Deng, Jisheng Wang, Xudong Yu, Xuefeng Gong, Yanfeng Li, Hongwei Yuan

**Affiliations:** a Department of Andrology; b The Third Affiliated Hospital of Beijing University of Chinese Medicine; c Department of Urology; d Department of Acupuncture and Moxibustion, Dongzhimen Hospital, Beijing University of Chinese Medicine, Beijing, China.

**Keywords:** protocol, systematic review, traditional Chinese medicine, ureteral

## Abstract

**Background:**

Ureteral calculi generally refer to the temporary obstruction of the human body after the ureteral stenosis. When the ureteral stones are not discharged in time, they can grow in the original site, causing the patient to have corresponding clinical manifestations, such as: renal colic, hematuria, etc, when severe, can cause renal obstruction and hydronephrosis, seriously endangering the patient's health. Ureteral calculi usually occur in young and middle-aged people. The peak age of the disease is between 20 and 50 years old. It also occurs in the young and middle-aged labor force. The men incidence rate is 2 to 3 times that of women. Ureteral calculi is one of the current refractory diseases, and the effect after treatment with integrated Chinese and Western medicine is remarkable.

**Methods and analysis:**

We will search for PubMed, Cochrane Library, AMED, EMbase, WorldSciNet; Nature, Science online and China Journal Full-text Database (CNKI), China Biomedical Literature CD-ROM Database (CBM), and related randomized controlled trials included in the China Resources Database. The time is limited from the construction of the library to November 2018. We will use the criteria provided by Cochrane 5.1.0 for quality assessment and risk assessment of the included studies, and use the Revman 5.3 and Stata13.0 software for meta-analysis of the effectiveness, recurrence rate, and symptom scores of ureteral.

**Ethics and dissemination:**

This systematic review will evaluate the efficacy and safety of Traditional Chinese medicine for ureteral. Because all of the data used in this systematic review and meta-analysis has been published, this review does not require ethical approval. Furthermore, all data will be analyzed anonymously during the review process Trial.

Registration number: PROSPERO CRD42019137095

## 1 INTRODUCTION

Ureteral calculi are products of abnormal accumulation of crystalline substances and organic matter in the urinary system, which may be related to water quality and dietary structure.^[1,2]^ Ureteral calculi are generally caused by kidney stones during the discharge process, temporarily blocked in the stenosis of the ureter.^[3–6]^ Primary ureteral stones are rare. Young and middle-aged people are high-risk groups: the peak age of onset is 20 to 50 years old, which is the labor force that occurs in the prime of life, in which men are 2 to 3 times more likely than women.^[7]^ If the ureteral stones are not discharged, they may gradually grow up at the resting part. Ureteral stones are usually accompanied by obvious symptoms:

(1) Lumbar cramps: Renal colic is a typical symptom of ureteral stones, usually after the exercise or at night, one side of the back and back severe pain, because it is too painful to describe the knife Cut the sample, at the same time can appear in the lower abdomen and inner thigh pain, nausea and vomiting, pale, and so on.^[8]^ The patient is restless and very painful. Some patients present with dull pain and pain in the lower back.^[9]^ After the pain, some patients can find stones that are excreted with the urine.
(2) Hematuria: About 80% of patients have hematuria, and only a part of them can be found by the naked eye to be red, and most of them can only be found by laboratory tests.^[10]^
(3) Asymptomatic: Many patients accidentally found ureteral stones during physical examination without any symptoms.^[11]^
(4) Hydronephrosis: Stones block the ureter, urine discharge is not smooth, causing hydronephrosis.^[12]^ Some hydronephrosis can be without any symptoms. Long-term hydronephrosis can cause impaired kidney function in the affected side. Severe bilateral hydronephrosis may cause uremia.^[13]^
(5) Fever: Ureteral stones can also induce bacterial infection, leading to kidney empyema, high fever.^[14]^ Because the stones hinder the discharge of urine, the bacteria cannot be discharged in time, and in severe cases, sepsis can be caused and life-threatening.^[15]^

At present, the more popular technology is to use extracorporeal vibrating stone technology to remove stones, but many treatments will leave a small amount of stones, and the removal is not complete, so that the remaining stones have signs of recurrence.^[16]^

Compared with modern treatment, traditional Chinese medicine has great advantages in treating urinary stones. For example, traditional Chinese medicine contains more active ingredients and has various pharmacological effects. Moreover, Chinese medicine attaches importance to the overall concept and dialectically uses traditional Chinese medicine.^[17]^ It not only saves patients from the pain of surgery, but also reduces adverse reactions and recurrence rates.^[18]^ The clinical treatment methods of traditional Chinese medicine are various, mainly including heat-clearing and damp-discharging method, Zhuangyao Jianshen method, Wenshen Lishui method, Yiqi method, Yangyin method, promoting blood circulation and removing blood stasis method, and relieving pain relief method.^[19]^ Traditional Chinese medicine treatment of kidney stones and ureteral stones is still a clinically effective and inexpensive treatment method. There are many advantages that cannot be replaced by surgical treatment, which has been recognized and promoted by clinicians.

## 2 Methods

This systematic review protocol has been registered on PROSPERO CRD42019137095 (http://www.crd.york.ac.uk/PROSPERO/display_record.php?ID=CRD42019137095).The protocol follows the Cochrane Handbook for Systematic Reviews of Interventions and the Preferred Reporting Items for Systematic Reviews and Meta-Analysis Protocol (PRISMA-P) statement guidelines. We will describe the changes in our full review if needed.

### 2.1 Inclusion criteria for study selection

#### 2.1.1 Types of studies

We will gather all studies of Traditional Chinese medicine on treating ureteral calculi: a systematic review and meta-analysis which, no matter whether they have been published or not, base on the method of randomized controlled trial (RCT). The language is limited to Chinese and English. Non-RCTs quasi-RCTs, series of case reports, and cross research will be excluded.

#### 2.1.2 Types of participants

Adults diagnosed with ureteral calculi have been included, which means there are no restrictions on age, region, country, ethnicity, and source.

#### 2.1.3 Types of interventions

The drug composition, the dose-sepididymitiscific Chinese medicine preparation, or the combined western medicine are used as exepididymitisrimental interventions. Both prescription and Chinese patent medicines will be included. Other traditional Chinese medicine treatments such as intravenous medication, TCM, and massage will be limited.

##### 2.1.3.1 Control interventions

As for control intervention, people receiving virtual Chinese medicine treatment can be used as a placebo control or without any treatment, as a blank control will be used. However, once they receive Chinese medicine treatment or other Chinese medicine treatment, the trial will be rejected.

The following treatment comparisons will be investigated:

(1) Chinese medicine and no treatment;
(2) Chinese medicine and placebo/false Chinese medicine;
(3) Chinese medicine and modern medical drug treatment;
(4) Chinese medicine and other active treatment;

#### 2.1.4 Types of outcome measures

##### 2.1.4.1 Primary outcomes

The main criteria are: symptoms disappeared; no abnormal urine results; renal pyelography showed no stones.

##### 2.1.4.2 Secondary outcomes

Secondary evaluation criteria included: reduced use of painkillers; reduced incidence of renal colic; reduced recurrence rate. At the same time, close attention should be paid to whether adverse reactions or adverse events occur during the experiment to comprehensively evaluate the clinical efficacy and safety of traditional Chinese medicine in the treatment of ureteral calculi.

### 2.2 Search methods for the identification of studies

#### 2.2.1 Electronic searches

Database Search: Search PubMed, Cochrane, Library, AMED, EMbase, WorldSciNet; Natural Science Online and China National Knowledge Infrastructure (CNKI), China Biomedical Disc (CBMdisc). The time interval is limited from the time the database is created to May 2019, and a combination of keywords and free word retrieval is used. Search terms include “Chinese medicine,” “Ureteral calculi.” The search term in the Chinese database is the translation of the above words. The complete PubMed search strategy is summarized in Table 1.

**Table 1:**
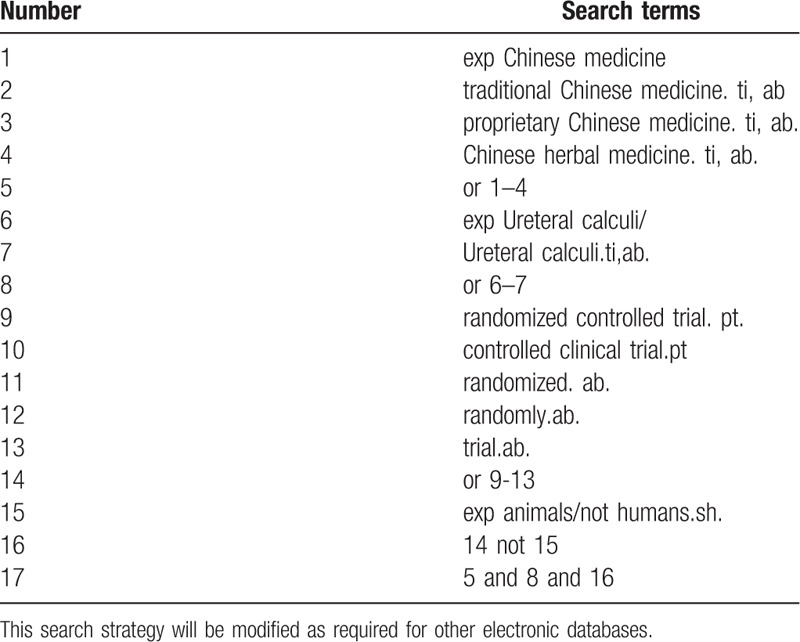
Search strategy used in PubMed database.

#### 2.2.2 Searching other resources

Manual search for relevant literature, earlier than the above databases, such as “China Journal of Rehabilitation Medicine,” “Chinese Journal of Physical Medicine and Rehabilitation,” and “Chinese Journal of Urology.”

### 2.3 Data collection and analysis

#### 2.3.1 Study identification

**Figure 1:**
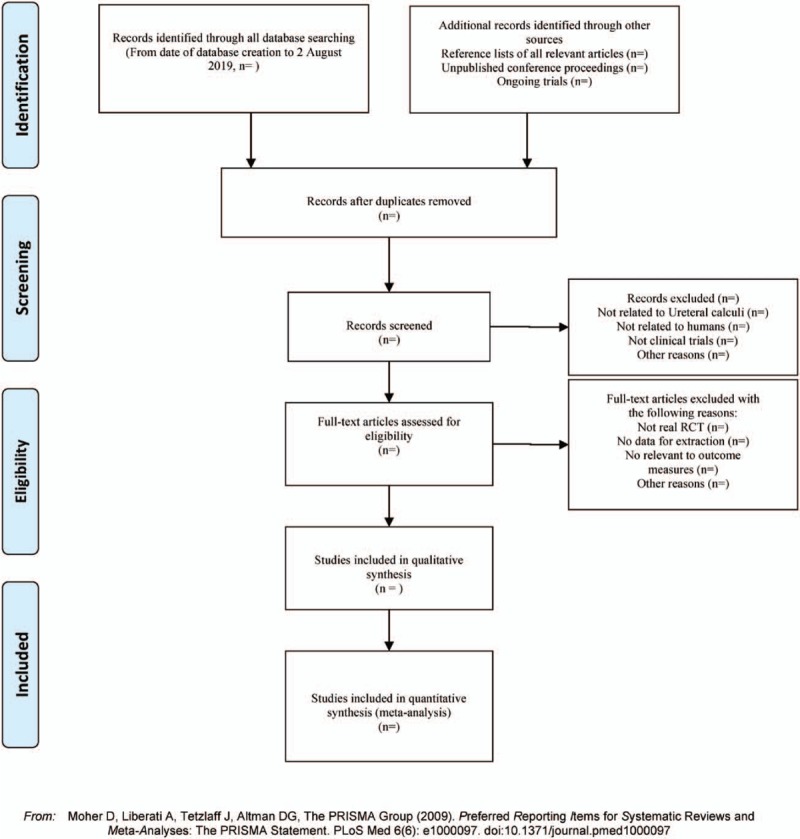
The PRISMA flow chart. PRISMA-P = Preferred Reporting Items for Systematic Reviews and Meta-Analyses.

(1) There are 2 researchers filtering out the literature that clearly do not conform to the study such as meeting minutes dissertations reviews animal experiments and so on, which, after excluding all the retrieved documents from the duplicated literature, adopt the method of reading the title of the literature abstracts etc. The details of selection process will be shown in the PRISMA flow chart (Fig. 1).
(2) The second time of screening the literature: skimming the remaining documents and filtering out unqualified documents such as case reports theoretical discussions and non-conformance of interventions.
(3) The third time of screening the literature: carefully reading the remaining documents and strictly filtering out unqualified documents such as general controlled trials, lacking control group, deficiency of random allocation, incompatible outcome indicator, and the appearance of similar data etc.
(4) As for the literature that cannot be ensured, it would be confirmed by the discussion of the 2 researchers. And if they cannot reach an agreement, the third-party experts would get involved, which aims at absorbing the appropriate RCTs into the study.

#### 2.3.2 Data extraction and management

The data extraction of the literature will be done independently by the 2 researchers, filling in the data format developed by the researchers. The data extraction content includes the following:

(1) General information: article title, first author, correspondent author, publication study time, evaluation letter, contact information.
(2) Research methods: design patterns, adequate scale, random assignment, random hiding, blinding, baseline levels.
(3) Participants: patient age, sex, diagnostic criteria for ureteral stones, severity, ethnic studies, location.
(4) Intervention: Chinese medicine, treatment period, treatment times.
(5) Efficacy evaluation: The main observation indicators were the safety indicators of the secondary observation indicators and the number of adverse reactions.
(6) Note: source of funds, medical ethics review, important reference materials.

#### 2.3.3 Assessment of risk of bias in included studies

As for the Literature quality evaluation, we will use the bias risk assessment tool recommended by Cochrane to assess the quality of all included literature and risk of bias. The assessment include: sequence generation; allocation concealment; blinding of participants, personnel, and outcome assessors; incomplete outcome data; selective outcome reporting; other sources of bias. The evaluation above would be independently evaluated by 2 researchers. If there are different opinions, we discuss them. If there are still differences exist, we would consult the third appraiser. Otherwise, we need to consult with the Cochrane Professional Group for solution.

#### 2.3.4 Statistical analysis

The meta-analysis studied in this review will adopt Rev Man5.3 and Stata13.0 statistical software. Heterogeneity test will be used for the inclusion of the study, and random or fixed effect models will be adopted, with *P* < .05 as the test standard. If the heterogeneity between the results is too large, the random effects model (REM), which deduces the source of heterogeneity by sensitivity analysis, will be used for the rest analysis. Secondly, according to the different type of statistical data, the binary categorical variable will use the odds ratio (OR) and its 95% confidence interval (CI) as the effect analysis index. As for the continuous variable, the standardized mean difference (SMD) and its 95% CI will be used as the effect analysis index. If the outcome measures only provide the means and standards deviation before or after treatment, the Mean_change_ and the SD_change_ are obtained according to the method provided in Cochrane Handbook 5.1.0: 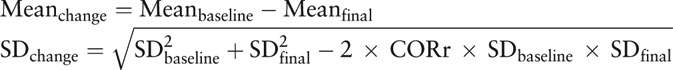


The forest map and funnel plot were drawn and analyzed using Rev Man5.3 software, and the funnel plot was used to analyze potential publication bias. As for the Literature quality evaluation, we will use the bias risk assessment tool recommended by Cochrane to assess the quality of all included literature and risk of bias. The assessment include: sequence generation; allocation concealment; blinding of participants, personnel, and outcome assessors; incomplete outcome data; selective outcome reporting; other sources of bias. The evaluation above would be independently evaluated by 2 researchers. If there are different opinions, we discuss them. If there are still differences exist, we would consult the third appraiser. Otherwise, we need to consult with the Cochrane Professional Group for solution.

#### 2.3.5 Assessment of heterogeneity

We will use a chi-square test to estimate heterogeneity of both the MD and OR. Further analysis can be performed using the *I*^2^ test. If possible, we will also construct a forest plot for analysis. A random-effect model will be used to interpret the results if heterogeneity is statistically significant, whereas a fixed-effect model will be used if heterogeneity is not statistically significant. We will regard heterogeneity as substantial when *I*^2^ is >50% or a low *P* value (<.10) is reported for the chi-square test for heterogeneity.^[16]^

#### 2.3.6 Sensitivity analysis

We will conduct a sensitivity analysis to identify whether the conclusions are robust in the review according to the following criteria: sample size, heterogeneity qualities, and statistical model (random-effects or fixed-effects model).

#### 2.3.7 Publication bias

If a result of a meta-analysis contains >10 articles and above, we will use a funnel plot to test the risk of publication bias.

#### 2.3.8 Quality of evidence

The quality of evidence for the main outcomes will also be assessed with the GRADE approach. The evaluation included bias risk; heterogeneity; indirectness; imprecision; publication bias. And each level of evidence will be made “very low,” “low,” “erate,” or “high” judgment.

## 3 Discussion

In recent years, clinical randomized controlled trials of ureteral calculi have been increasing, but still unsatisfactory in the diagnosis and treatment of diseases.^[20]^ Clinicians have not yet reached a consensus on the principles and assessment of the treatment of the disease, and there is no uniform standard of standardization.^[21]^ There has not been a large-scale epidemiological investigation of the disease, and there are few reports in the literature. Traditional Chinese medicine treatment of ureteral stones has a profound theoretical foundation and rich clinical experience. Although the specific mechanism of traditional Chinese medicine treatment of ureteral calculi is not very clear, but clinical studies have shown that Chinese medicine treatment of ureteral stones can discharge stones to a certain extent, relieve pain, and improve symptoms. To the best of our knowledge, there is no comparison of the effectiveness of traditional Chinese medicine in the treatment of ureteral stones.^[22,23]^

Therefore, we will use systematic reviews and meta-analyses to assess the efficacy and safety of traditional Chinese medicine in the treatment of ureteral calculi.^[24]^ The results of this study can provide a possible ranking for Chinese medicine treatment of ureteral calculi.^[25]^ In addition, the scoring method will be used to assess the quality of evidence for the primary outcome. We hope that these results will provide clinicians with the basis for the treatment of ureteral calculi with traditional Chinese medicine and provide the best choice for the treatment of patients.^[26]^ In addition, although this study will conduct a comprehensive search, it will not search for languages other than Chinese and English, which will lead to some bias.

## Author contributions

**Conceptualization:** Haisong Li, Sheng Deng, Yanfeng Li.

**Data curation:** Yanfeng Li, Hongwei Yuan.

**Funding acquisition:** Haisong Li.

**Investigation:** Sheng Deng, Xudong Yu.

**Methodology:** Xudong Yu.

**Project administration:** Xudong Yu, Hongwei Yuan.

**Supervision:** Jisheng Wang, Xuefeng,Gong.

**Validation:** Jisheng Wang, Xuefeng Gong.

**Visualization:** Xuefeng Gong.

**Writing – review & editing:** Xuefeng Gong.
